# Association Between Cancer Stem Cell Marker Expression and Clinicopathological Features in Thai Patients With Hepatocellular Carcinoma

**DOI:** 10.7759/cureus.89761

**Published:** 2025-08-10

**Authors:** Supreecha Chattong, Pattama Wongsirisin, Chanaporn Raman, Oraya K Ngamlert

**Affiliations:** 1 Department of Medical Research and Technology Assessment, National Cancer Institute, Thailand, Bangkok, THA; 2 Department of Anatomical Pathology, National Cancer Institute, Thailand, Bangkok, THA; 3 Department of Clinical Pathology and Medical Technology, Lerdsin Hospital, Bangkok, THA

**Keywords:** cancer stem cells, cd133, cd44, cd90, epcam, hepatocellular carcinoma

## Abstract

Introduction

Hepatocellular carcinoma (HCC) is a leading cause of cancer-related mortality worldwide. Cancer stem cells (CSCs) are believed to play critical roles in tumor initiation, progression, metastasis, and treatment resistance. This study aimed to evaluate the expression of the four most commonly CSCs with relevant to HCC, namely, CD44, CD90, CD133, and epithelial cell adhesion molecule (EpCAM), in tumor and adjacent noncancerous tissues of Thai HCC patients and to explore their association with clinicopathological features and patient survival.

Methods

HCC patients undergoing curative hepatic resection at the National Cancer Institute, Thailand, were included. Tumor and matched adjacent noncancerous tissues were collected and analyzed for the expression of CD44, CD90, CD133, and EpCAM using immunohistochemistry (IHC). IHC scores were calculated based on staining intensity and extent. Associations between marker expression and clinicopathological features were assessed, and overall survival was analyzed using the Kaplan-Meier method.

Results

All four CSC markers were significantly overexpressed in tumor tissues compared to adjacent normal tissues (P < 0.05). However, no statistically significant associations were found between marker expression and clinicopathological parameters. Kaplan-Meier analysis showed no significant differences in overall survival between high and low expression groups. Nonetheless, patients with low CD44 and CD133 expression tended to have better survival outcomes, although not statistically significant.

Conclusion

The findings suggest that CD44, CD90, CD133, and EpCAM were enriched in HCC tumor tissues, supporting their role in hepatocarcinogenesis. However, no strong correlations were found with clinical features or survival in this cohort.

## Introduction

Hepatocellular carcinoma (HCC) is the sixth most commonly diagnosed cancer worldwide and the third leading cause of cancer-related mortality. The high incidence and mortality rates associated with liver cancer make it a critical global public health concern [1]. Early-stage HCC is often asymptomatic, and diagnosis typically occurs at an advanced stage with tumor progression or metastasis. Even in cases where the tumor is detected early and surgically resected, more than 70% of patients experience recurrence. The five-year survival period for liver cancer patients is typically limited to three to 13 months after treatment. Therefore, it characterized by poor prognosis and limited treatment efficacy [2,3].

Primary tumor consists of genetically diverse cancer cell populations; tumor heterogeneity includes a subpopulation of cells known as cancer stem cells (CSCs). These cells possess stem-like properties, including self-renewal and the ability to differentiate into tumor-initiating cells. CSCs play essential roles in tumor growth, metastasis, recurrence, and resistance to chemotherapy [4]. CSC markers have been identified in various malignancies including HCC [5-7].

In HCC, several CSCs have been identified such as CD44, CD90, CD133, and epithelial cell adhesion molecule (EpCAM), and these cells are expressed as liver CSCs (LCSCs). Many studies have shown that the expression of such markers is associated with increased tumorigenic capacity and metastatic potential and correlates with larger tumor size, early recurrence, chemotherapy resistance, and reduced survival in HCC patients [8-20]. The present study aimed to analyze the expression of CD44, CD90, CD133, and EpCAM in tumor tissue and paired adjacent normal tissue from Thai HCC patients undergoing curative surgical resection who had not previously received therapy to search for associations with clinicopathological characteristics and survival time.

## Materials and methods

Patients and HCC samples

Fifteen cases of HCC patients who were diagnosed and treated at the National Cancer Institute, Thailand, between January 2020 and December 2022 were included in this study. The inclusion criteria were as follows: Thai patients and age over 40 years, first HCC diagnosis, and no previous therapies. The exclusion criteria were extrahepatic metastasis and other concurrent malignancies. This study was approved by the Research Ethics Committee, National Cancer Institute, Thailand, based on the Declaration of Helsinki and Good Clinical Practice (EC67007). Clinicopathological data were collected, such as age at diagnosis, sex, histological differentiation, and tumor size.

HCC tumor samples

Single HCC lesion and surrounding adjacent normal tissues were collected. The tissue samples were fixed in 10% formalin for 24 hours and then processed. Processed tissues were embedded in paraffin, cut into 4 𝜇m thick sections, and stained by hematoxylin and eosin (H&E). The sample representative areas were identified and marked. For immunohistochemical staining, the sections were mounted onto adhesive glass slide (Platinum Pro, Osaka, Japan).

Immunohistochemistry (IHC) and scoring

Immunohistochemistry (IHC) for anti-CD133 (EPR16508), anti-CD90 (EPR3133), anti-CD44 (EPR1013Y), and anti-EpCAM (EPR20532-225) was performed to detect the in situ expression. All antibodies were purchased from Abcam (Cambridge, UK). Initially, 4 𝜇m thick of paraffin-embedded tissue sections on adhesive glass slides were deparaffinized with xylene and rehydrated with a series of graded ethanol. Slides were retrieved antigens using 1% zinc sulfate solution by boiling in a microwave for 30 minutes and then quenched endogenous peroxidase activity with 3% hydrogen peroxide for 10 minutes. The non-specific binding sites were blocked with 1% bovine serum albumin solution. Each primary antibody against human CD133, CD90, CD44, and EpCAM was stained at room temperature for one hour, followed by secondary antibody conjugated with horseradish peroxidase enzyme at room temperature for 30 minutes. Staining was detected with 2,3-diaminobenzidine tetrahydrochloride for 10 minutes and counterstained with hematoxylin. All histological slides were examined by light microscopy (Olympus BX51, Tokyo, Japan). The IHC results were determined as described previously [9]. The expression levels were scored in two parts. The positive extent score was determined using a percentage of positive stained cell (0-100%), and the staining intensity was evaluated using a scale of 0-3 as negative, weak, moderate, or strong (0, 1, 2, and 3, respectively). The IHC score was calculated by multiplying the positive extent score with the staining intensity score. The thresholds for high versus low expression were determined based on evaluations by two independent pathologists who were blinded to patient clinical data. Discrepancies were resolved by consensus. An IHC score higher than mean score was determined as high expression, while a score lower than or equal to mean score was defined as low expression.

Statistical analysis

Data were expressed as mean ± standard deviation (SD) or relative risk ratio. The continuous data comparisons were performed using student’s t-test, while the categorical data comparisons were performed using the Chi-square test or Fisher exact test. Survival curves were performed using the Kaplan-Meier method, and differences between curves were determined by the log-rank test. P < 0.05 was considered statistically significant.

## Results

Demographic and clinicopathological characteristics in HCC patients

A total of 15 patients were included in this study. The majority were male (66.7%), while females accounted for 33.3% with no statistically significant difference (P = 0.1965). Most patients were aged 50 years or older (93.3%), and age was significantly associated with the studied variable (P = 0.0078). Regarding tumor characteristics, 66.7% of patients had a single tumor, whereas 33.3% had multiple tumors (P = 0.1965). A statistically significant association was observed with tumor size; 86.7% of patients had tumors smaller than 5 cm, while only 13.3% had tumors ≥5 cm (P = 0.0045). For tumor differentiation, 40.0% of patients had well-differentiated tumors, and 60.0% had moderate to poorly differentiated tumors, with no significant difference observed (P = 0.4389). Vascular invasion was absent in most cases (73.3%), while 26.7% of patients presented with vascular invasion. This difference was statistically significant (P = 0.0078). Finally, 66.7% of patients had cirrhosis, whereas 33.3% did not, with no statistically significant difference (P = 0.1965) (Table 1).

**Table 1:** Demographic and clinicopathological characteristics in hepatocellular carcinoma (HCC) patients The categorical data comparisons were performed using the Chi-square test. P < 0.05 was considered statistically significant.

Variables	Data (Number (%))	P-value
Sex		0.1965
Female	5 (33.3)	
Male	10 (66.7)	
Age (year)		0.0078
<50	1 (6.7)	
≥50	14 (93.3)	
Tumor number		0.1965
Single	10 (66.7)	
Multiple	5 (33.3)	
Tumor size		0.0045
<5 cm	13 (86.7)	
≥5 cm	2 (13.3)	
Tumor differentiation		0.4389
Well	6 (40.0)	
Moderate/Poor	9 (60.0)	
Vascular invasion		0.0078
Absent	11 (73.3)	
Present	4 (26.7)	
Cirrhosis		0.1965
Absent	5 (33.3)	
Present	10 (66.7)	

Expression of liver CSC markers CD44, CD90, CD133, and EpCAM in tumor and paired noncancerous adjacent tissues

**Figure 1:**
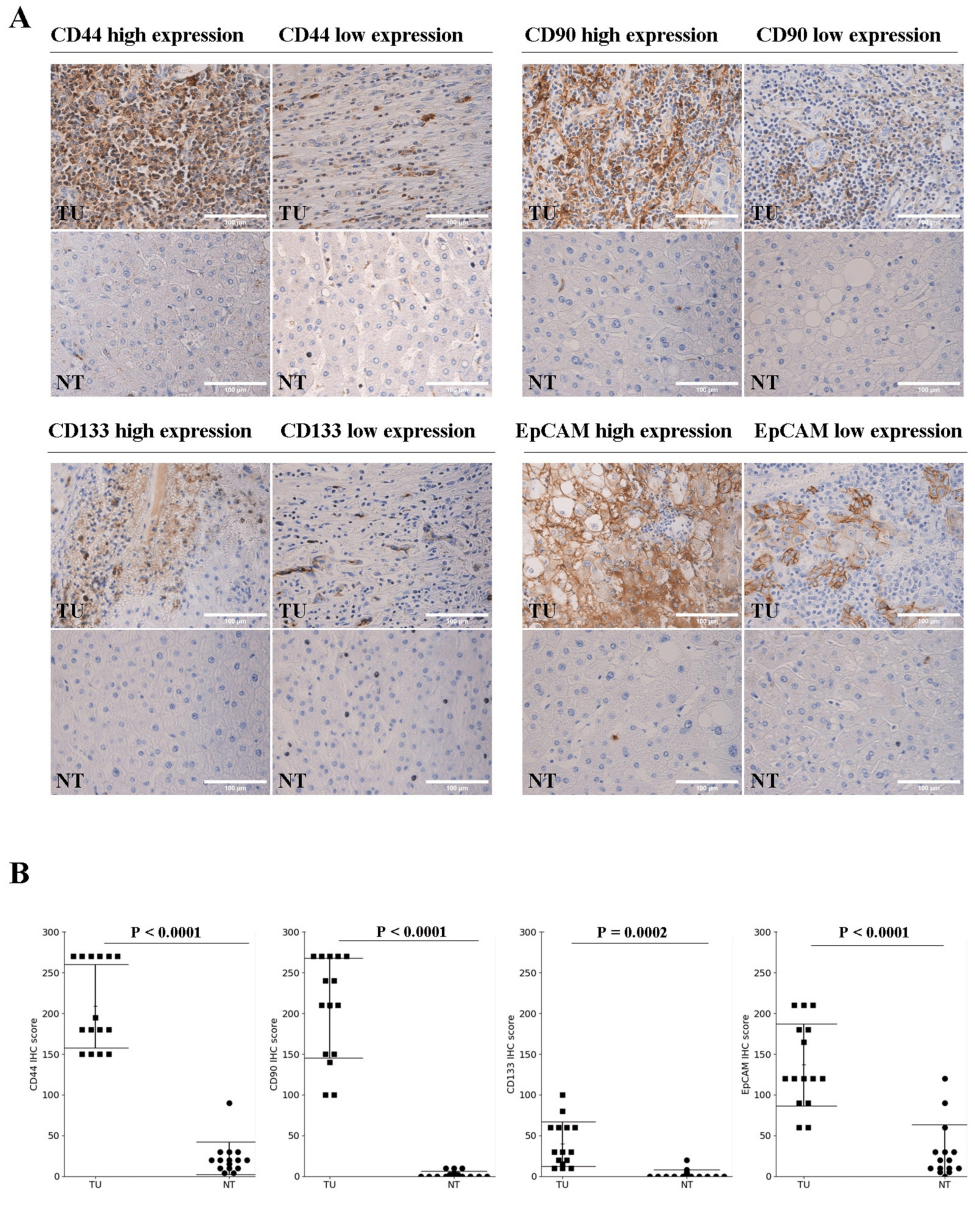
Immunohistochemistry of the expression of CD44, CD90, CD133, and EpCAM in HCC tissue (TU) and normal adjacent tissue (NT). (A) Representative images of CD44, CD90, CD133, and EpCAM staining in HCC and normal adjacent tissue (magnification 40x). (B) Expression analysis of CD44, CD90, CD133, and EpCAM in HCC and normal adjacent tissue. The continuous data comparisons were performed using Student’s t-test. P < 0.05 was considered statistically significant.

Fifteen pairs of tumor tissues and corresponding adjacent normal tissues were collected from HCC patients undergoing hepatic surgical resection at the National Cancer Institute, Thailand. Immunohistochemistry analysis revealed that protein was mainly expressed in cytoplasm of tumor cells for CD44, CD90, and CD133, while EpCAM was mostly expressed in cell membrane. The expression levels of CD44, CD90, CD133, and EpCAM were evaluated in HCC tumor compared with corresponding non-tumor tissues, as shown in Figure 1A. Statistical analysis of IHC scores for each marker revealed significantly higher expression of all four markers CD44, CD90, CD133, and EpCAM in tumor tissues compared to non-tumor tissues (P < 0.05), as shown in Figure 1B.

Association of clinicopathological characteristics with CD44, CD90, CD133, and EpCAM expression in HCC patients

The expression levels of CD44, CD90, CD133, and EpCAM were analyzed in relation to various clinicopathological variables, including tumor number (single or multiple), tumor size (<5 cm or ≥5 cm), tumor differentiation (well or moderate to poor), vascular invasion (absent or present), and cirrhosis (absent or present). Among all these variables, no statistically significant associations were observed between the HCC characteristics and the expression of any of the four markers (CD44, CD90, CD133, and EpCAM) (all P-values > 0.05). However, CD44 expression tended to be higher in HCC patients with cirrhosis (P = 0.0889), although this did not reach statistical significance (Table 2).

**Table 2:** Association of clinicopathological characteristics with CD44, CD90, CD133, and EpCAM expression in HCC patients The categorical data comparisons were determined using the Fisher's exact test. There were no statistical differences between the HCC characteristics and the expression of any of the four markers (CD44, CD90, CD133, and EpCAM) (P-values > 0.05).

Variables	No.	CD44		P-value	CD90		P-value	CD133		P-value	EpCAM		P-value
		Low	High		Low	High		Low	High		Low	High	
Tumor number				1			1			1			1
Single	10	6	4		3	7		6	4		6	4	
Multiple	5	3	2		2	3		3	2		3	2	
Tumor size				1			1			0.4857			0.4857
<5 cm.	13	8	5		4	9		7	6		7	6	
≥5 cm	2	1	1		1	1		2	0		2	0	
differentiation				0.3287			0.5804			1			0.2867
Well	6	3	3		1	5		3	3		5	1	
Moderate/poor	9	7	2		4	5		5	4		4	5	
Vascular invasion				0.6044			0.5604			1			1
Absent	11	6	5		3	8		7	4		7	4	
Present	4	3	1		2	2		2	2		2	2	
Cirrhosis				0.0889			1			0.5804			0.5804
Absent	5	1	4		2	3		4	1		4	1	
Present	10	8	2		3	7		5	5		5	5	

Association of overall survival with CD44, CD90, CD133, and EpCAM expression in HCC patients

**Figure 2:**
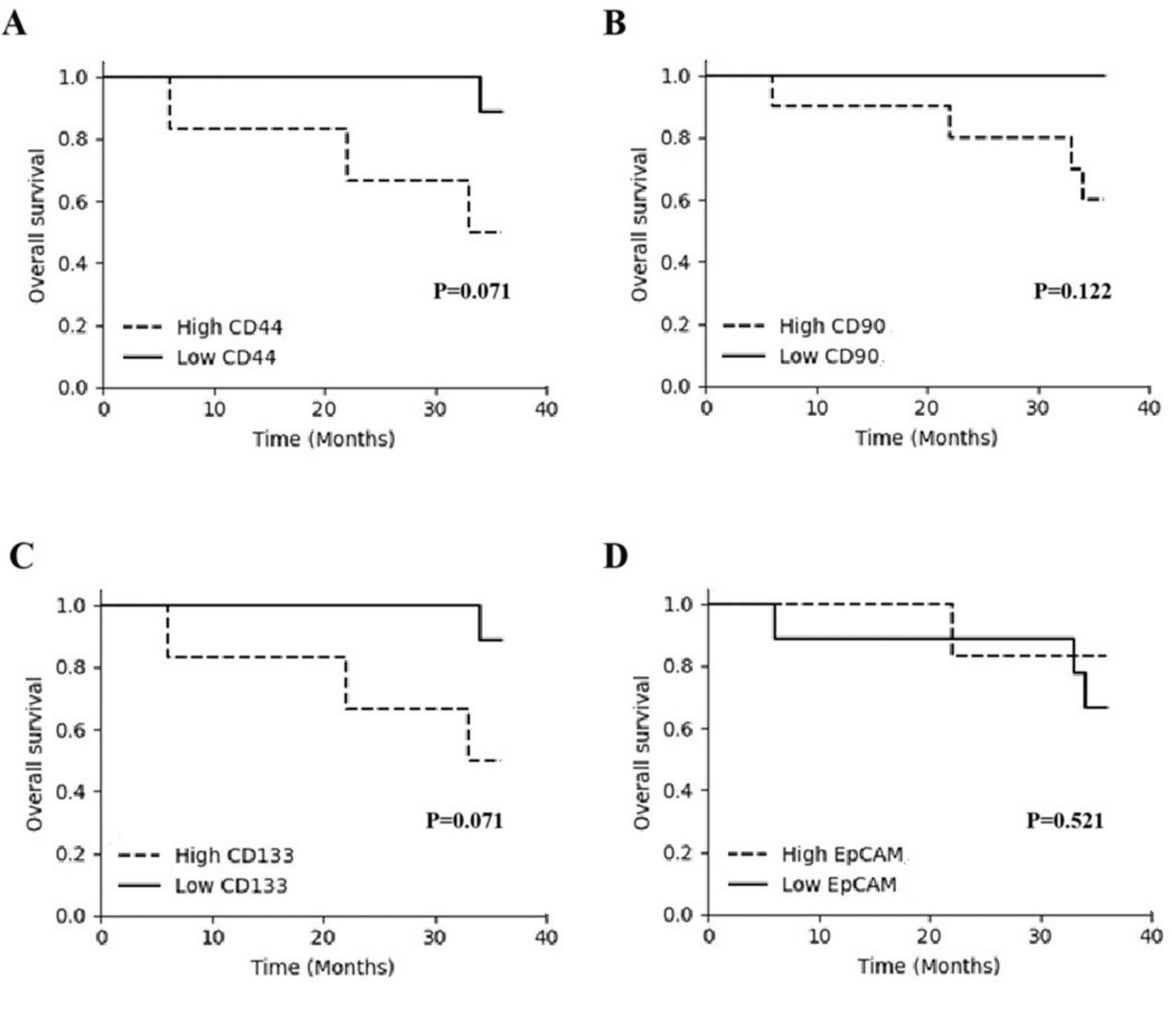
Overall survival of HCC patients with high or low expression of (A) CD44 (P = 0.071), (B) CD90 (P = 0.122), (C) CD133 (P = 0.071), and (E) EpCAM (P = 0.521) Survival curves were performed using the Kaplan-Meier method and differences between curves were determined by the log-rank test. There were no statistically significant differences in overall survival between high- and low-expression groups for CD44, CD90, CD133, or EpCAM patients (P > 0.05).

Kaplan-Meier survival analysis was performed to assess the prognostic impact of CSC marker expression on overall survival. As shown in Figure 2, there were no statistically significant differences in overall survival between high- and low-expression groups for CD44, CD90, CD133, or EpCAM patients (P > 0.05). HCC patients with low CD44 expression tended to have better overall survival compared to those with high CD44 expression, particularly beyond 20 months. However, the difference did not reach statistical significance (P = 0.071; Figure 2A). A similar trend was observed for CD133 expression, where low CD133 expression was associated with better survival (P = 0.071; Figure 2C). By contrast, there were no significant differences in overall survival between the high- and low-expression groups for CD90 (P = 0.122; Figure 2B) and EpCAM (P = 0.521; Figure 2D).

## Discussion

Many evidence have demonstrated that CSCs, which possess stem-like properties, including self-renewal and the ability to differentiate into tumor-initiating cells play essential roles in tumor growth, metastasis, recurrence, and resistance to chemotherapy [6-8]. CSC markers have been identified in various malignancies, including lung, pancreatic, breast, prostate, colorectal, glioma, and liver cancers [9-13]. In this study, we investigated the correlation of the four most commonly CSCs, namely, CD44, CD90, CD133, and EpCAM, with the relevance of HCC features.

CD44, a cell surface glycoprotein, acts as a receptor for hyaluronic acid, mediating various intracellular signaling pathways [21]. CD44 has been identified in multiple cancers, including breast, prostate, colorectal, and pancreatic [22-25]. In HCC, CD44 expression is recognized as a key LCSC marker. CD44 gene knockout in overexpressing HCC cells reduces CSC properties, enhances chemosensitivity, and inhibits cell migration and invasion in vivo. CD44 expression has been correlated with aggressive tumor behavior and epithelial-mesenchymal transition (EMT) in HCC patients after surgical resection [8-10]. Notably, HCC cells co-expressing CD44 and CD133 exhibit enhanced stemness, self-renewal, tumorigenic potential, and chemoresistance [11].

CD90 also known as Thy-1, is another critical CSC marker in HCC. CD90 is a glycosylphosphatidylinositol (GPI)-anchored protein found in various cell types, including thymocytes, T-cells, neurons, endothelial cells, and fibroblasts [26]. CD90 contributes to tumorigenicity and metastasis in multiple HCC cell lines and has been used to isolate LCSCs from both tumor tissues and patient blood samples [9,10]. When CD90+ cells are injected into immunodeficient mice, they can initiate tumor formation. CD90 expression is associated with increased tumorigenic capacity and metastatic potential and correlates with larger tumor size, early recurrence, and reduced survival in HCC patients [13,14].

CD133 or prominin-1 is a cell surface glycoprotein. It was initially discovered in CD34+ hematopoietic stem cells and has since been found in brain, pancreatic, prostate, and colorectal cancers [27-29]. In HCC, CD133 was first isolated from the Huh7 cell line. CD133+ cells exhibit rapid proliferation, tumorigenic potential, and low expression of mature hepatocyte markers [30]. Clinically, high CD133 expression in HCC correlates with poor prognosis, lower survival rates, and high recurrence [15]. CD133 is implicated in multiple molecular mechanisms, including self-renewal, differentiation, tumor initiation, and chemoresistance [16,17].

EpCAM is a membrane glycoprotein expressed in epithelial tissues and various cancers. It has been identified on liver and pancreatic CSCs. Transplantation of EpCAM⁺/CD45⁻ cells into immunodeficient mice induces tumor formation, whereas EpCAM⁻/CD45⁻ cells do not [18]. EpCAM⁺ cell populations are regulated via the Wnt/β-catenin signaling pathway. Overexpression of EpCAM and β-catenin in Dt81 hepa1-6 cells increases tumor formation. Clinical studies have shown persistent EpCAM expression in HCC patients even after curative tumor resection, suggesting a role in recurrence [18,19]. EpCAM is significantly overexpressed in HCC tissues compared to adjacent normal liver, and its high expression is associated with advanced clinical stage, poor differentiation, and shorter survival [20].

Our finding revealed that significantly higher expression of all four studied markers CD44, CD90, CD133, and EpCAM in HCC tumor tissues compared to corresponding non-tumor tissues (P < 0.05). This finding supports evidences suggesting that these markers are enriched in cancerous tissues and play a role in hepatocarcinogenesis. Interestingly, despite the elevated expression of CSC markers in tumor tissues, no significant associations were observed between marker expression and clinicopathological characteristics such as tumor number, size, differentiation, vascular invasion, or cirrhosis. This may reflect the complex and heterogeneous nature of CSC marker expression in HCC, which could be influenced by genetic background, tumor microenvironment, or etiology. Notably, CD44 expression tended to be higher in patients with cirrhosis, consistent with reports linking CD44 to liver fibrogenesis and chronic liver injury [9].

In the survival analysis, although not statistically significant, there was a trend toward better overall survival in patients with low CD44 and CD133 expression. This observation supports the hypothesis that high expression of these markers may be associated with aggressive tumor behavior and poorer prognosis. Several studies have identified CD44 and CD133 as independent prognostic indicators in HCC, associated with increased tumor invasiveness and decreased survival [9-11]. The lack of statistical significance in our cohort may be due to the small sample size, underscoring the need for validation in larger populations.

Overall, our findings reinforce the relevance of CSC markers in HCC biology. While their diagnostic and prognostic utility remains under investigation, these markers may serve as potential therapeutic targets. Targeting CSCs has emerged as a promising strategy to overcome treatment resistance and reduce recurrence in HCC. Finally, this study had limitations include the small sample size and single-center design, which may limit the generalizability of the findings. Future studies with larger cohorts and functional validation are needed to elucidate the mechanistic roles of these markers and their clinical utility in HCC management.

## Conclusions

Our study provides insights into the demographic and clinicopathological landscape of HCC and highlights the elevated expression of CD44, CD90, CD133, and EpCAM in tumor tissues. While these markers did not show strong associations with conventional clinicopathological features or overall survival in this cohort, the observed trends, particularly for CD44 and CD133, warrant further investigation. Future research with larger patient cohorts is crucial to fully elucidate the roles of these cancer stem cell markers in HCC progression and their potential as prognostic or therapeutic targets.
